# Clinical characteristics of 4,520 paediatric patients infected with the SARS-CoV-2 omicron variant, in Xi'an, China

**DOI:** 10.3389/fped.2024.1325562

**Published:** 2024-02-23

**Authors:** Jingwei Yue, Jin Cao, Lin Liu, Li Yin, Mingyue Li

**Affiliations:** ^1^ Department of Emergency, Xi’an Children's Hospital (Xi'an Jiaotong University Affiliated Children’s Hospital), Xi'an, Shaanxi, China; ^2^ Department of Gastroenterology, Xi'an Tus-Children’s Hospital, Xi'an, Shaanxi, China

**Keywords:** omicron, coronavirus, SARS-CoV-2, COVID-19, acute necrotizing encephalopathy

## Abstract

**Background and objective:**

Severe acute respiratory syndrome coronavirus 2 (SARS-CoV-2) has broad tissue tropism and high transmission, which are likely to perpetuate the pandemic. The study aim to analyze the clinicopathogenic characteristics in paediatric patients.

**Methods:**

In this single-centre study, we retrospectively included all confirmed cases infected by SARS-CoV-2 infection at Xi’an Children's Hospital, China, from 1 December to 31 December 2022. The demographic, clinical, laboratory, and radiological features of the patients were analysed.

**Results:**

A total of 4,520 paediatric patients with SARS-CoV-2 omicron variant infections were included. Of these, 3,861 (85.36%) were outpatients, 659 (14.64%) were hospitalised patients, and nine patients (0.20%) died. Of the nine patients who died, five were diagnosed with acute necrotising encephalopathy (ANE). The most common symptoms were fever in 4,275 (94.59%) patients, cough in 1,320 (29.20%) patients, convulsions in 610 (13.50%) patients, vomiting in 410 (9.07%) patients, runny nose/coryza in 277 (6.13%) patients, hoarseness of voice in 273 (6.04%) patients. A blood cell analysis showed a slight elevation of monocytes (mean: 11.14 ± 0.07%). The main diagnoses for both outpatients and inpatients were respiratory infection with multisystem manifestations.

**Conclusions:**

A high incidence of convulsions is a typical characteristic of children infected with SARS-CoV-2. Five of the nine COVID-19 fatalities were associated with ANE. This indicates that nervous system damage in children with SARS-CoV-2 infection is more significant.

## 1 Introduction

Severe acute respiratory syndrome coronavirus 2 (SARS-CoV-2) has caused a permanently pandemic of acute respiratory disease (1, 2). “Omicron”, a new variant of the Coronavirus (B.1.1.529) has led to a global pandemic (3). These new BQ and XBB sub-variants of Omicron are transmitted far more rapidly than other variants of SARS-CoV-2 (4–6). SARS-CoV-2 is highly transmissible with broad tissue tropism that is likely perpetuating the pandemic (7). The infection of humans with SARS-CoV-2, lead to severe “flu”-like symptoms that can progress to pneumonia, acute respiratory distress, renal failure, and death (8–10). This study aimed to investigate the clinical characteristics of a large sample of paediatric patients infected with the omicron variant who were diagnosed and treated at our hospital.

## 2 Materials and methods

### 2.1 Patient selection

This retrospective, observational, single-centre study was conducted from 1 December to 31 December 2022. A total of 4,520 paediatric patients were infected with the omicron variant at Xi’an Children's Hospital (The Affiliated Children's Hospital of Xi'an Jiaotong University) in Xi'an, China. All patients enrolled in this study were diagnosed according to World Health Organization interim guidance (11). Throat­swab specimens from the upper respiratory tract that were obtained from all patients at admission were maintained in viral­transport medium. 2019­nCoV was confrmed by real­time RT­PCR. The omicron variant was confirmed by whole-genome sequencing performed by the China Disease Control and Prevention Agency. The demographic and clinical characteristics and laboratory test results of the patients were analysed. This study was approved by the Institutional Review Board of the Affiliated Children's Hospital of Xi'an Jiaotong University.

### 2.2 Clinical data collection

We collected information from the electronic medical records of paediatric patients who tested positive for SARS-CoV-2 nucleic acids. The demographic data included age, weight, and sex. All the patients were from China. The clinical data included diagnosis, clinical signs, symptoms, and treatment. Among them, convulsions, maximum body temperature, and fever duration upon admission were highlighted. The laboratory data included a full blood cell count, hypersensitive C-reactive protein (hs-CRP), liver function, and myocardial enzymes.

### 2.3 Statistical analysis

Statistical analysis was performed using SPSS software (version 22.0). Continuous variables were described using the mean, median, and range. Additionally, categorical variables were described as frequency rates and percentages.

## 3 Results

### 3.1 Demographic and clinical data

A total of 4,520 paediatric patients with SARS-CoV-2 Omicron variant infections were included in this study. Of these, 3,861 (85.36%) were outpatients, 659 (14.64%) were hospitalised patients, and 9 (0.20%) patients died. The proportion of males was 58.14% and females was 41.86%. The age of onset ranged from newborns to 18 years of age; patients aged 1–3 years accounted for the main proportion. The median patient age was 2.08 (0–18) years (Table 1).

**Table 1:** Demographics and clinical characteristics of 4,520 patients infected with the omicron variant.

Characteristics	No. (%) of patients	Median
Age group (years)		2.08 (0.00–17.75)
Neonatal patients	167 (3.69%)	
<1	1,191 (26.35%)	
1–3	1,451 (32.10%)	
3–6	844 (18.67%)	
6–12	735 (16.26%)	
12–18	132 (2.92%)	
Sex		
Male (*n*, %)	2,628 (58.14%)	
Female (*n*, %)	1,892 (41.86%)	
Death rate (*n*, %)	9 (0.20%)	
Hospitalized patients	659 (14.58%)	
Outpatients	3,861 (85.42%)	
Initial presenting symptoms		
Fever time upon admission (hour)		24 (0–600)
No fever	245 (5.42%)	
Headache	61 (1.35%)	
Cough	1,320 (29.20%)	
Sore throat	121 (2.68%)	
Hoarseness of voice	273 (6.04%)	
Abdominal pain	57 (1.26%)	
Vomiting	410 (9.07%)	
Runny nose/coryza	277 (6.13%)	
Myalgia	5 (0.11%)	
Fatigue/weakness	20 (0.44%)	
Convulsion	610 (13.50%)	
Initial presenting clinical signs		
Congestion of the pharynx	all	
Pharyngeal secretions	18 (0.40%)	
Enlarged tonsils		
Ⅰ°	61 (1.35%)	
Ⅱ°	60 (1.33%)	
Ⅲ°	6 (0.13%)	
Coarse breath sounds	525 (11.62%)	
With crackles	56 (1.24%)	
With crackles and wheezing	58 (1.28%)	
Rash	24 (0.53%)	

On admission, most patients had fever. Other common symptoms included cough, convulsion, vomiting, runny nose/coryza, hoarseness of voice, sore throat, muscle ache, or headache. The initial presenting clinical signs were mainly involved in respiratory system. The initial presenting symptom was fever in 4,275 patients (94.59%); the median fever time upon admission to the emergency was 24 (0–600) hours. The most common symptoms were cough in 1,320 (29.20%) patients, convulsion in 610 (13.50%) patients, vomiting in 410 (9.07%) patients, runny nose/coryza in 277 (6.13%) patients, hoarseness of voice in 273 (6.04%) patients, sore throat in 121 (2.68%) patients, headache in 61(1.35%) patients, abdominal pain in 57 (1.26%) patients, myalgia in 5 (0.11%) patients, fatigue/weakness in 20 (0.44%) patients, and. The initial presenting clinical signs were congestion of the pharynx in all patients, rash in 24 (0.53%) patients, pharyngeal secretions in 18 (0.40%) patients, enlarged tonsils I° in 61(1.35%) patients, enlarged tonsils Ⅱ° in 60 (1.33%) patients, enlarged tonsils Ⅲ° in 6 (0.13%) patients, coarse breath sounds in 525 (11.62%) patients, patients with crackles 56 (1.24%), and patients with crackles and wheezing 58 (1.28%) (Table 1).

### 3.2 Laboratory data

Blood cell analysis was performed in all patients. There was no typical features except the percentage of monocytes was slightly elevated. The mean white blood cell count was 7.11 ± 0.05 (0.19–48.20) × 10^9^; of these, five patients with leukaemia were excluded with a white blood cell count of 118.32–338.72 × 10^9^. The mean percentage of lymphocytes was 31.84 ± 0.28 (1.86–88.8)%; the percentage of neutrophils was 55.63 ± 0.31 (0.60–94.60)%. The percentage of monocytes was 11.14 ± 0.07 (0.00–46.6)%. The mean value of high-sensitivity C-reactive protein was 8.14 ± 0.29 (0.02–664.0) mg/L (Table 2).

**Table 2:** Laboratory characteristics of paediatric patients infected with the omicron variant.

Blood cell analysis and hs-CRP of all patients (*n* = 4,520)		
	Mean	Range
White blood cell (×10^9^)	7.11 ± 0.05	0.19–48.20
Percentage of lymphocytes (%)	31.84 ± 0.28	1.86–88.8
Percentage of neutrophils (%)	55.63 ± 0.31	0.60–94.60
Percentage of monocytes (%)	11.14 ± 0.07	0.00–46.6
high-sensitivity C-reactive protein (mg/L)	8.14 ± 0.29	0.02–664.0
Of 659 inpatients, ALT levels of 118 patients (17.91%) and AST levels of 323 patients were elevated.		
	Mean	Range
ALT (U/L)	133.77 ± 307.02	35–2,251
AST (U/L)	116.30 ± 373.34	44–5,307
Of 659 inpatients, CK-MB of 305 patients (46.28%) and procalcitonin of 167 patients (25.34%) was elevated.		
	Mean	Range
Creatine kinase isoenzyme (U/l)	45.35 ± 45.10	25–535
Procalcitonin (ng/ml)	6.80 ± 16.85	0.5–134.95
Blood gas analysis in 42 patients of 659 inpatients		
pH	7.33 ± 0.23	6.63–7.49
Lactic acid (mmol/L)	2.08 ± 0.37	0.50–12

ALT, alanine aminotransferase; AST, aspartate aminotransferase; CK-MB, Creatine kinase isoenzyme.

Liver function, myocardial enzyme, procalcitonin were often elevated in severe cases,. The normal ranges for alanine aminotransferase (ALT) is 7–35 U/L, aspartate aminotransferase (AST) is 14–44 U/L, creatine kinase isoenzyme is 0–25 U/L, and procalcitonin is 0–0.5 ng/dl. Of 659 inpatients, ALT levels of 118 patients (17.91%) and AST levels of 323 patients were elevated. The mean ALT value was 133.77 ± 307.02 (35–2,251) U/L, AST was 116.30 ± 373.34 (44–5,307) U/L. Of 659 inpatients, creatine kinase isoenzyme (CK-MB) of 305 patients (46.28%) and procalcitonin of 167 patients (25.34%) was elevated. The mean CK-MB was 45.35 ± 45.10 (25–535) U/L; procalcitonin levels were 6.80 ± 16.85 (0.5–134.95) ng/ml. Blood gas analyses were performed in 42 patients. The mean of pH was 7.33 ± 0.23 (6.63–7.49) and lactic acid (LAC) was 2.08 ± 0.37 (0.50–12) mmol/L (Table 2).

### 3.3 Characteristics of main diagnoses

#### 3.3.1 Characteristics of main diagnoses in outpatients

**Figure 1:**
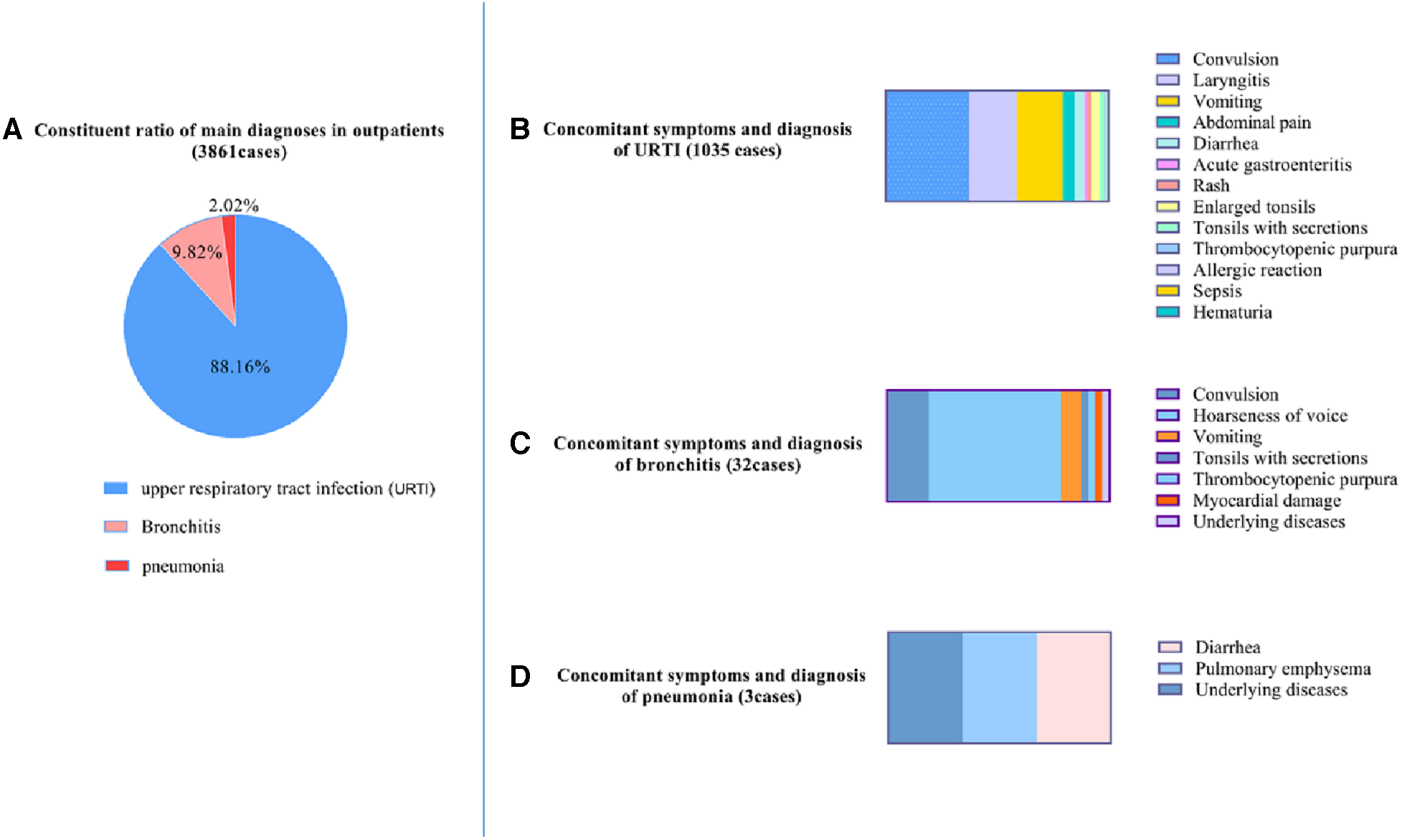
Characteristics of main diagnoses and concomitant symptoms in outpatients. (**A**) Constituent ratio of main diagnoses in outpatients; (**B**) upper respiratory tract infection (URTI) with concomitant symptoms. (**C**) Bronchitis with concomitant symptoms. (**D**) Pneumonia with concomitant symptoms.

Upper respiratory tract infection (URTI) was the main diagnosis about the outpatients and accompanied with other manifestations of various system damage, particularly convulsion. Of the 3,861 outpatients, 3,404 patients (88.16%) were diagnosed with upper respiratory tract infection, 379 patients (9.82%) with bronchitis, and 78 patients (2.02%) with pneumonia (Figure 1A). Of the patients with URTI, 1,035 patients presented with concomitant symptoms, such as convulsion (386 cases, 37.29%), laryngitis (224 cases, 21.64%), vomiting (214 cases, 20.68%), abdominal pain (55 cases, 5.31%), diarrhoea (46 cases, 4.44%), acute gastroenteritis (11 cases, 1.06%), rash (21 cases, 2.03%), enlarged tonsils (39 cases, 3.77%), tonsils with secretions (20 cases, 1.93%), thrombocytopenic purpura (6 cases, 0.58%), allergic reaction (2 cases, 0.19%), sepsis (2 cases, 0.19%), haematuria (1 case, 0.10%), and underlying diseases (8 cases, 0.77%) (Figure 1B). Among the bronchitis patients, 32 patients presented with concomitant symptoms such as convulsions (6 cases, 18.75%), hoarseness of voice (19 cases, 59.38%), vomiting (3 cases, 9.38%), tonsils with secretions (1 case, 3.13%), thrombocytopenic purpura (1 case, 3.13%), myocardial damage (1 case, 3.13%) (Figure 1C), and underlying diseases (1 case, 3.13%). Of the patients with pneumonia, 3 patients presented with concomitant symptoms, such as diarrhoea (1 case, 33.33%), pulmonary emphysema (1 case, 33.33%), and underlying diseases (1 case, 33.33%) (Figure 1D).

#### 3.3.2 Characteristics of main diagnoses in inpatients

**Figure 2:**
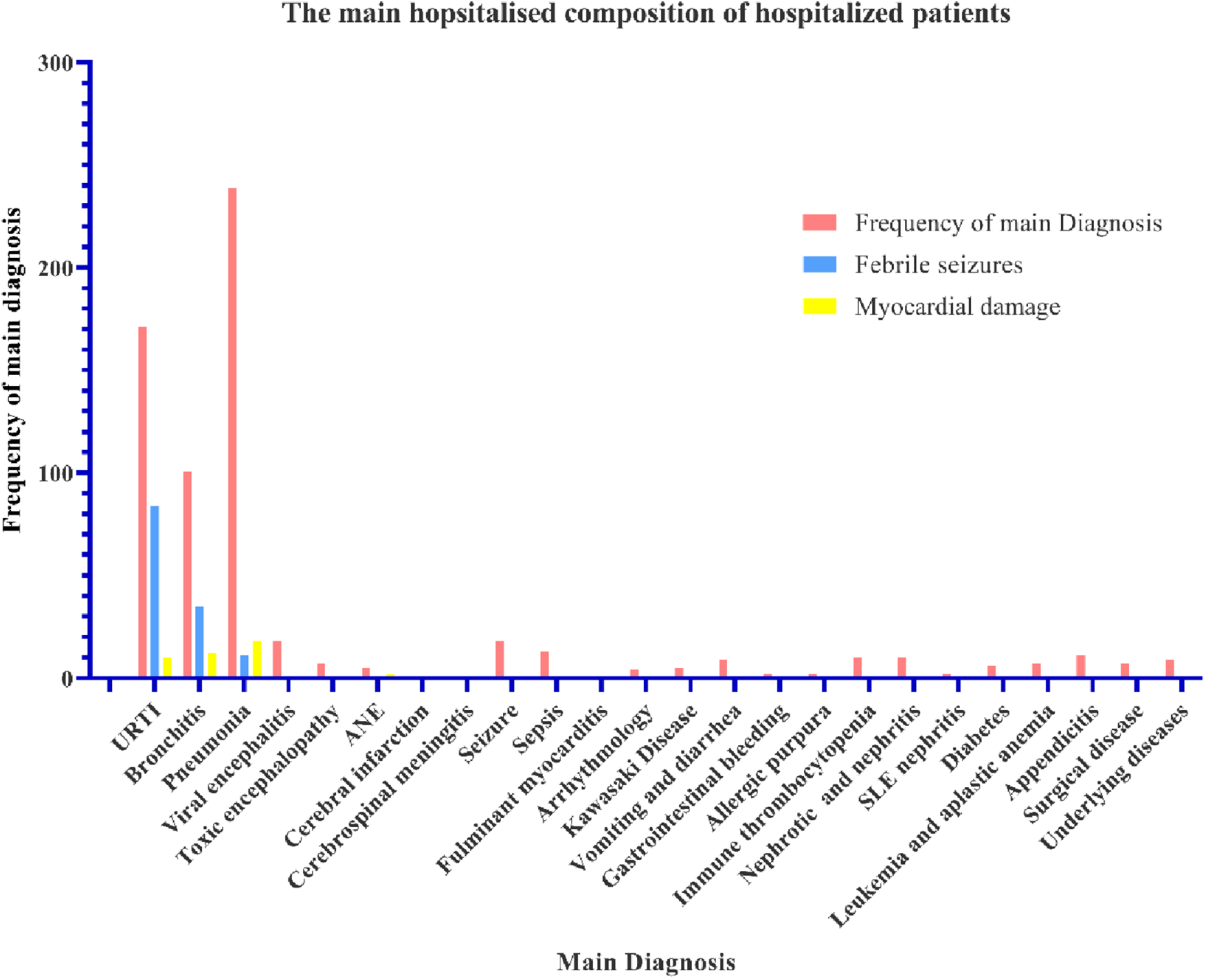
The main diagnostic composition of hospitalised patients. The abscissa is the diagnosis and accompanying symptoms. The ordinate is the frequency of the patients.

Pneumonia, URTI and bronchitis were the main diagnosis about the inpatients accompanied with multiple organ damage. Of the 659 inpatients, 171 patients (25.95%) were diagnosed with URTI; 101 patients (15.33%) had bronchitis; 239 patients (36.27%) had pneumonia (Figure 2); 18 had viral encephalitis; 7 had toxic encephalopathy; 5 had acute necrotising encephalopathy (ANE); 1 had cerebral infarction; 1 had cerebrospinal meningitis; 18 had seizures; 13 had sepsis; 1 had fulminant myocarditis; 1 had arrhythmology; 5 were diagnosed with Kawasaki disease; 9 had vomiting and diarrhoea; 2 had gastrointestinal bleeding; 2 had allergic purpura; 10 patients had immune thrombocytopenia; 10 patients were nephrotic and had nephritis; 2 had systemic lupus erythematosus (SLE) nephritis; 2 patients had diabetes; 7 had leukaemia and aplastic anaemia; 11 had appendicitis; 7 had surgical disease; and 9 patients had complicated underlying diseases. Febrile seizures occurred mainly in patients with URTI (84 cases, 49.12%), bronchitis (35 cases, 34.65%), and pneumonia (11 cases, 4.60%) (Figure 2). Myocardial damage also occurred mainly in URTI (10 cases, 5.85%), bronchitis (12 cases, 11.88%), and pneumonia (11 cases, 7.53%) (Figure 2).

#### 3.3.3 Characteristics of main diagnoses in patients who died

**Figure 3:**
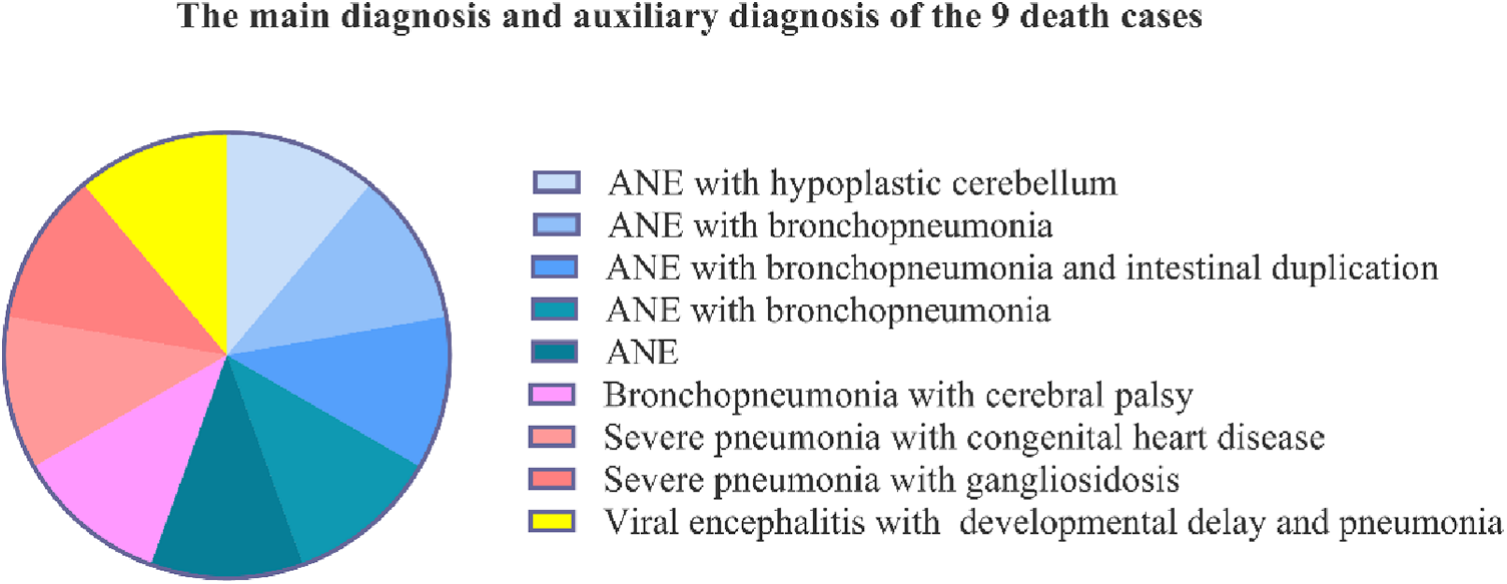
The main diagnosis of the 9 patients who died. ANE: acute necrotizing encephalopathy.

Of the nine patients who died, five were diagnosed with acute necrotising encephalopathy (ANE); two cases were associated with congenital malformations, which were hypoplastic cerebellum and intestinal duplication, and three cases were associated with bronchopneumonia. Of these patients, three were diagnosed with bronchopneumonia or severe pneumonia associated with cerebral palsy, congenital heart disease, and gangliosidosis, and one had viral encephalitis with developmental delay and pneumonia (Figure 3).

### 3.4 Clinical features of the patients who died (*n* = 9)

The main early symptoms and signs of ANE were high or very high fever, convulsions, and coma. The procalcitonin (PCT) level was obviously elevated, which is uncommon in viral infections. Of the nine patients who died, five were male and four were female. The median patient age was 1.92 (0.41–14.58) years. The main symptoms of pneumonia, were cough, cyanosis, phlegm, wet rales, and wheezing (Table 3). The blood cell analysis results were unremarkable. High-sensitivity C-reactive protein (CRP) levels were elevated in only one case of severe pneumonia. However, the procalcitonin (PCT) level was obviously elevated in six patients (mean: 41.74 ± 15.30). The pH was also markedly abnormal in six patients (mean: 7.22 ± 0.09). Blood lactic acid (LAC) was obviously elevated in five patients (mean: 4.62 ± 1.81), alanine aminotransferase (ALT) in four patients (mean: 493.78 ± 254.97), aspartate aminotransferase (AST) in seven patients (mean: 1,042.78 ± 582.48), and creatine kinase isoenzyme (CK-MB) in five patients (mean: 102.33 ± 26.65) (Table 4).

**Table 3:** Clinical characteristics of 9 patients infected with the omicron variant who died.

Patient	Sex	Age	Main symptoms and signs	Highest temperature of fever	Main diagnosis
1	Male	14.58	Fever, convulsions, coma, phlegm	39.6	ANE with hypoplastic cerebellum
2	Male	1.33	Fever, convulsions, coma, wet rales	42	ANE with bronchopneumonia
3	Female	8.67	Fever, cyanosis, coma, wet rales	39.8	Bronchopneumonia with cerebral palsy
4	Female	0.41	Cough, asthma, low spirits, phlegm and wheeze, heart murmur	No fever	Severe pneumonia with congenital heart disease
5	Male	1.17	Fever, stiff limbs, coma, high muscle tone	40.5	ANE with bronchopneumonia and intestinal duplication
6	Female	1.92	Fever, cyanosis, coma, cyanosis	42	ANE with bronchopneumonia
7	Male	3.08	Fever, cough, Phlegm	39.1	Severe pneumonia with gangliosidosis
8	Female	1.25	Fever, cyanosis, coma, Papanicolaou sign positive	40.3	ANE
9	Male	2.00	Cyanosis, coma, phlegm, decreased muscle strength	No fever	Viral encephalitis with developmental delay and pneumonia

ANE, acute necrotizing encephalopathy.

**Table 4:** Blood analysis of the 9 patients infected with the omicron variant who died.

Patient	WBC	N%	L%	CRP	PCT	pH	LAC	ALT	AST	CKMB
1	7.99	74.5	17.9	0.5	35.41	7.45	3.12	13	53	44
2	6.65	44.9	53.9	3.96	33.16	6.94	15.8	1,165	1,803	97
3	2.8	68.2	27.5	14.21	134.95	7.32	1.1	575	1,649	246
4	4.83	26.9	60.3	0.5	0.05	7.38	1.9	21	48	27
5	1.46	43.2	49.3	1.33	0.27	7.18	0.6	32	82	20
6	15.54	26.1	66.3	6.25	23.48	6.63	12	343	114	101
7	7.85	23.1	65.9	262.9	58.53	7.37	2.04	24	194	56
8	3.36	56.2	40.2	27.37	89.4	7.2	3.7	2,251	5,307	213
9	5.87	78	15.5	0.59	0.45	7.47	1.3	20	135	117
Mean	6.26 ± 1.38	49.01 ± 7.12	44.09 ± 6.61	35.29 ± 28.61	41.74 ± 15.30	7.22 ± 0.09	4.62 ± 1.81	493.78 ± 254.97	1,042.78 ± 582.48	102.33 ± 26.65

WBC, White blood cells; N%, percentage of neutrophils; L%, percentage of lymphocytes; CRP, high-sensitivity C-reactive protei; PCT, procalcitonin; LAC, blood lactic acid; ALT, alanine aminotransferase; AST, aspartate aminotransferase; CKMB, creatine kinase isoenzyme.

**Figure 4:**
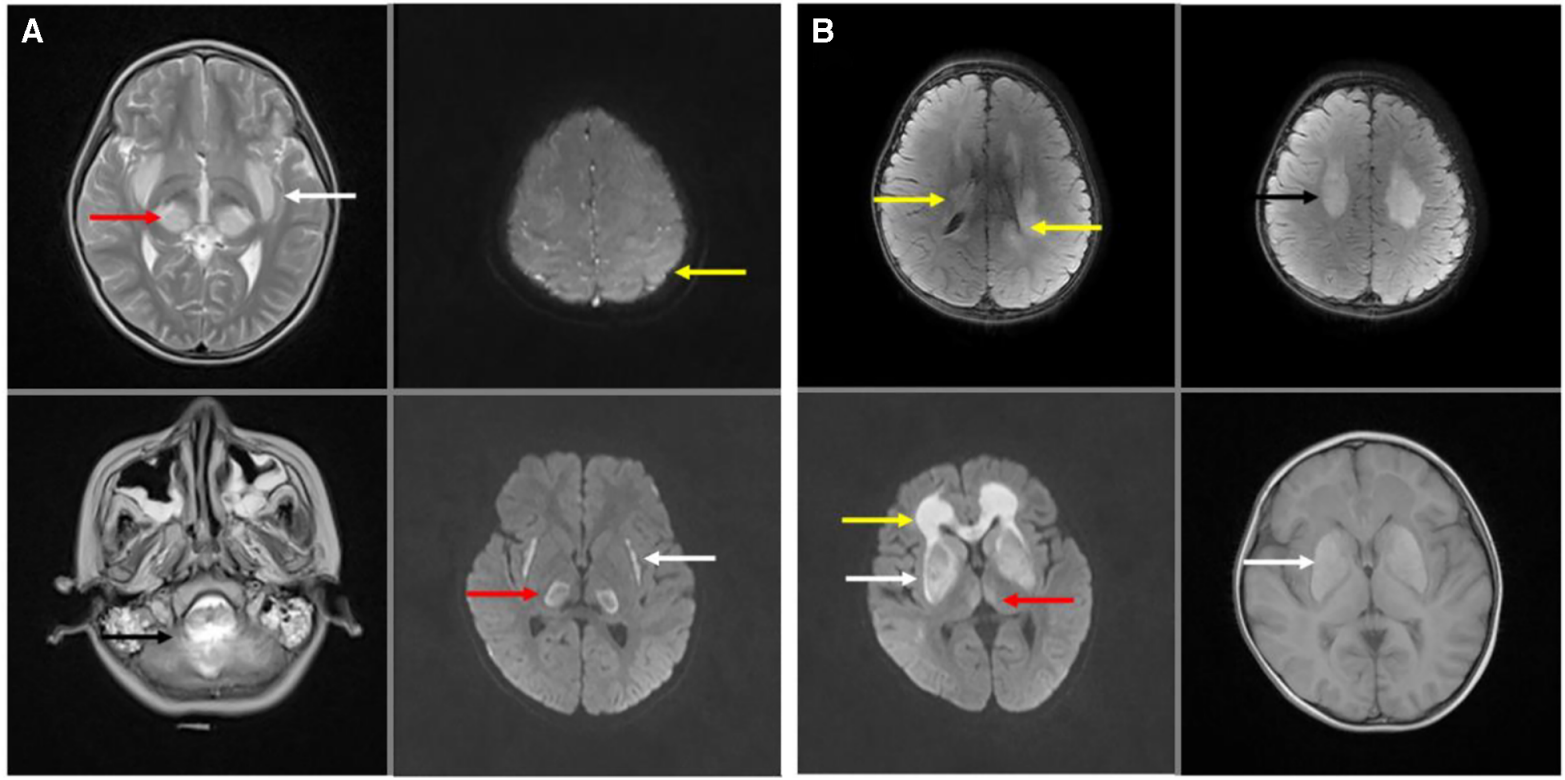
Brain nuclear magnetic resonance (MRI) of acute necrotizing encephalopathy. (**A**) Bilateral thalamic symmetrical damage in T2- weighted and T2-weighted flair (red arrow). Brain stem and cerebellum injured in T2- weighted (black arrow). The cerebral cortex was extensively oedematous with significant damage to the left parietal cortex (yellow arrow) in T2-weighted flair. External and internal capsule injury in T2-weighted flair (white arrow). (**B**) Bilateral thalamic symmetrical damage in T2-weighted flair (red arrow). Periventricular white matter damage in T2-weighted flair (yellow arrow). Centrum semiovale damaged in T2-weighted flair (black arrow). Basal ganglia damaged in T2-weighted flair and T1-weighted (white arrow).

In brain nuclear magnetic resonance imaging (MRI), bilateral thalamic symmetrical damage were observed in all patients with ANE. The typical lesion showed restricted diffusion in the thalami and swelling and increased T2-weighted signal (Figures 4A,B: red arrows). Symmetrical, multifocal thalamic damage is distinctive of ANE. It always involves the brainstem and cerebellum (Figure 4A: black arrow), and the parietal cortex (Figure 4A: yellow arrow), periventricular white matter, and the corpus callosum (Figure 4B: yellow arrow), and basal ganglia (Figure 4B: white arrow), and external and internal capsule injuries (Figure 4A: white arrow), and the centrum semiovale (Figure 4B: black arrow), temporal lobe, and amygdala. All patients had bilaterally symmetrical lesions, often showing as cytotoxic oedema, ischaemia, and necrotising changes in the lesion area.

### 3.4 Spearman's correlation analyses between lab findings with clinical diagnoses

**Figure 5:**
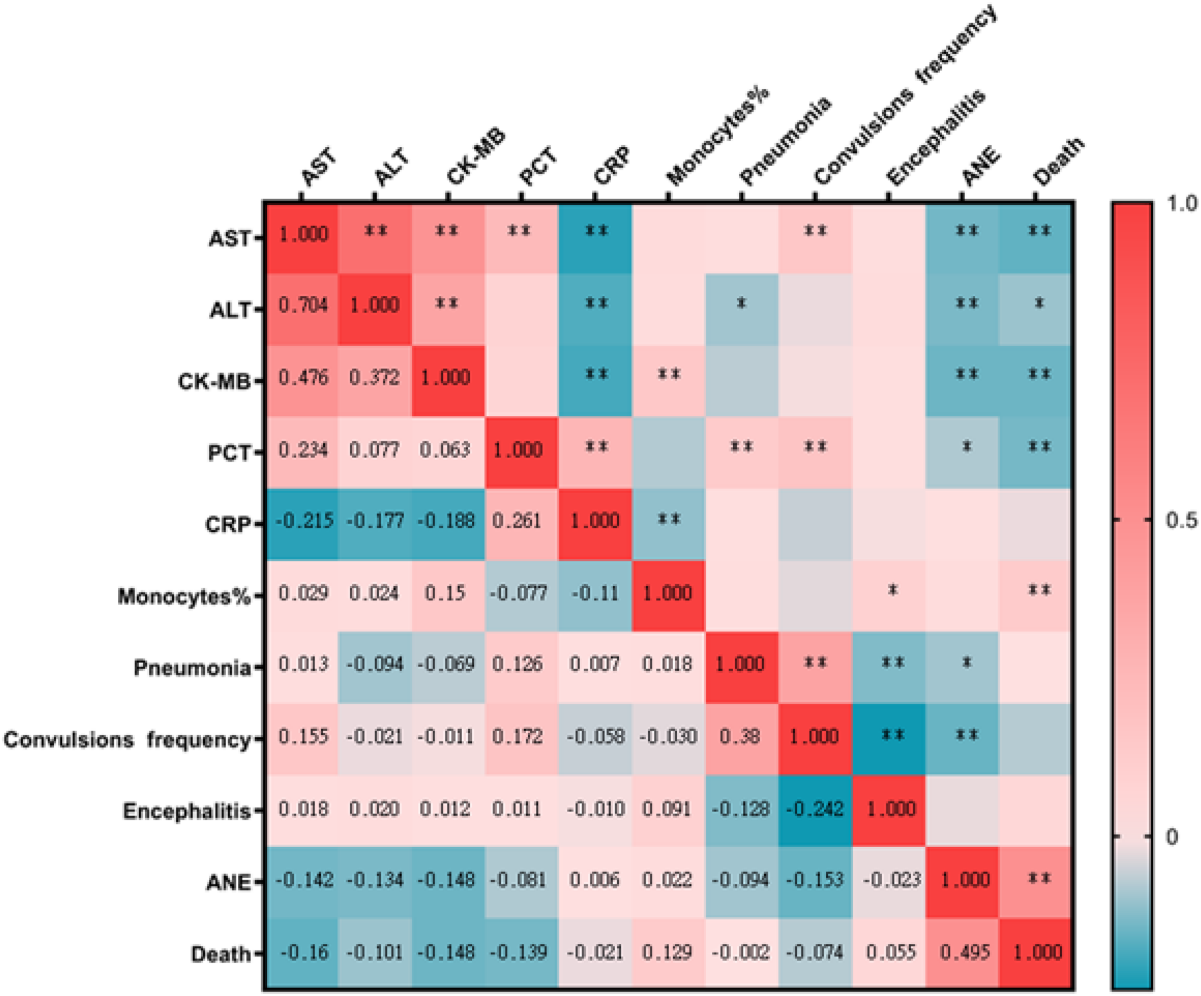
A heat map of Spearman's correlation analyses between lab findings with clinical diagnoses. *, correlation; **, Significant correlation. AST, aspartate aminotransferase; ALT, alanine aminotransferase; CK-MB, creatine kinase isoenzyme; PCT, procalcitonin; CRP, hypersensitive C-reactive protein; Monocytes%, percentage of monocytes; ANE, acute necrotizing encephalopathy.

Spearman's correlation coefficient was calculated to determine a statistically significant correlation between the lab findings with clinical diagnoses. AST and CK-MB were significant negatively correlated with ANE (AST: *r_s_* = −0.140, CK-MB: *r_s_* = −0.148, *p* < 0.001) and death (AST: *r_s_* = −0.160, CK-MB: *r_s_* = −0.148, *p* < 0.001). AST were significant positively correlated with frequency of convulsions (*r_s_ *= 0.155, *p* < 0.001). ALT were significant negatively correlated with ANE (*r_s_* = −0.134, *p *= 0.001) and negatively correlated with death (*r_s_* = −0.160, *p* = 0.01). PCT were significant negatively correlated with death (*r_s_* = −0.139, *p* = 0.001) and negatively correlated with ANE (*r_s_* = −0.081, p = 0.045). Percentage of monocytes were significant positively correlated with death (*r_s_* =0.129, *p* = 0.001). The correlation result show that children with SARS-CoV-2 Omicron Variant infection had higher levels of AST, ALT, CK-MB and PCT correlated with more risk of ANE and death. Elevated AST maybe was a predictors of convulsions. Elevated percentage of monocytes maybe was a protective factors of death (Figure 5).

### 3.5 Predictive factors for severe disease requiring hospitalization

A logistic analysis was performed. The results showed that fever time upon admission (hour), peak fever, WBC, neutrophils percentage, CRP and convulsion frequency were independent risk factors for hospitalization of children with Omicron Variant infection (*P* < 0.05). The odds ratio values were 1.022, 0.943, 1.072, 0.976, 1.005 and 2.844 respectively as shown in Table 5.

**Table 5:** Logistic analysis for the related factors predicting hospitalization of children with omicron variant infection.

Index	*β*	SE(*β*)	Wald	*p*	OR	95% CI
Gender	0.012	0.104	0.014	0.906	1.012	0.826, 1.240
Age	0.027	0.018	2.269	0.132	1.027	0.992, 1.063
Fever time	0.021	0.001	227.966	0.000	1.022	1.019, 1.024
Peak fever	−0.058	0.007	62.233	0.000	0.943	0.930, 0.957
WBC	0.070	0.014	25.367	0.000	1.072	1.044, 1.102
Monocytes%	0.010	0.010	0.966	0.326	1.010	0.990, 1.030
Neutrophils%	−0.024	0.009	6.663	0.010	0.976	0.958, 0.994
Lymphocytes%	−0.006	0.010	0.392	0.531	0.994	0.975, 1.013
CRP	0.005	0.002	5.209	0.022	1.005	1.001, 1.010
Convulsion	1.045	0.075	195.416	0.000	2.844	2.456, 3.292

WBC, white blood cells; CRP, hypersensitive C-reactive protein.

## 4 Discussion

Strong natural immunity is acquired after the primary infection by SARS-CoV-2 and may last for more than one year (12). However, Nguyen et al. reported that the severity of the second SARS-CoV-2 infection was similar to that of the first infection (13). Therefore, it is important to understand the pathogenic characteristics of SARS-CoV-2 in children. This was a descriptive study on the clinical characteristics of COVID-19, including data on 4,520 paediatric patients with Omicron variant infections. The mortality rate was 0.20% and the rate of hospitalization was 14.64%.

The aetiopathogenesis of SARS-CoV-2 infection in humans is reveals itself as mild symptoms to severe respiratory failure (14). In this study, 3,861 (85.36%) patients were outpatients with mild symptoms. The initial and main presenting symptom was a fever. The most common accompanying symptoms were sequent to cough (29.20%), convulsions (13.50%), vomiting (9.07%), hoarseness of voice (6.04%). The most typical symptom is a significant increase in convulsions. This indicates that nervous system damage in children with SARS-CoV-2 infection is more significant.

Of the nine patients who died, five were diagnosed with acute necrotising encephalopathy (ANE), of which the main symptoms and signs were high fever or extremely high fever, convulsions, and coma; two cases were associated with hypoplastic cerebellum and intestinal duplication; three cases were associated with bronchopneumonia; one was viral encephalitis with developmental delay and pneumonia. Six (66.67%) patients died of damage to the central nervous system. The pathogenesis is unknown. In a recent animal study, SARS-CoV-2 infections caused encephalitis in mouse models, which is not common in adult patients with SARS-CoV-2 (15–17), but was found in paediatric patients in our study. During early infection, viral replication occurs in multiple respiratory and non-respiratory tissues, including the brain (18). Varga et al. found evidence of direct SARS-CoV-2 infection of the endothelial cell by the ACE2 (angiotensin converting enzyme 2) receptor and diffuse endothelial inflammation (19, 20). It can also act as a receptor for SARS-COV-2, mediating viral entry into cerebral cells and spreading to the infective area (20). The SARS-COV-2 uses the ACE2 receptor expressed by alveolar epithelial cells to infect the host, causes lung injury, and recruits immune cells (19), which triggers a strong immune response known as cytokine storm syndrome (14). The “cytokine storm” can lead to widespread endothelial dysfunction and apoptosis in multiple organs (19). It is hypothesised that this mechanism also exists in cerebral vessels. When the cerebral vascular endothelial cells are injured, thrombus formation and occlusion can easily occur. In this study, all ANE cases showed ischaemia and necrotising changes in the basal ganglia, which is consistent with this mechanism. They often present as bilateral symmetrical lesions typically in the thalamus. The uniform and symmetrical distribution damage due to the energy depletion state (21, 22). This is a dynamic process that corresponds to clinical and pathophysiological changes, such as: cerebral edema-pitting, hemorrhage, necrosis-degenerative changes (23). Some patients may experience complete regression of the lesions. But others may have residual lesions such as atrophy, white matter cyst, hypothalamic density, and hemosiderin deposition (21, 24). Further studies are required to investigate the mechanisms of symmetrical damage.

Among the 659 inpatients, the main diagnoses were pneumonia (36.27%), URTI (25.95%), and bronchitis (15.33%). Common diagnoses were viral encephalitis, seizure, sepsis, appendicitis, immune thrombocytopenia, nephrosis and nephritis, vomiting and diarrhoea, complicated underlying diseases, encephalopathy, leukaemia and aplastic anaemia, surgical disease, ANE, Kawasaki disease. Rare diagnoses included gastrointestinal bleeding, allergic purpura, systemic lupus erythematosus, nephritis, diabetes, cerebral infarction, cerebrospinal meningitis, fulminant myocarditis, and arrhythmia. This indicates that decease easily lead to multiple organ dysfunctions (25, 26). Febrile seizures occurred mainly in URTI (84 cases, 49.12%), bronchitis (35 cases, 34.65%), and pneumonia (11 cases, 4.60%). Myocardial damage often occurred. The clinical characteristics of the disease include respiratory infections that can invade various organs and systems. A high incidence of febrile seizures is a typical characteristic of children infected with SARS-CoV-2. However, further studies are required to elucidate the underlying pathogenic mechanisms.

Of the 3,861 outpatients, the main diagnoses were URTI (88.16%), bronchitis (9.82%), and pneumonia (2.02%). Among the 1,035 patients with URTI, the patients presented with convulsion (37.29%). Among the 32 patients with bronchitis, the patients presented with convulsions (18.75%). The clinical characteristics of outpatients were respiratory infections with various systemic injuries and a high incidence rate of febrile seizures.

Upon comparison of adult patients with children with SARS-CoV-2 infections, the injuries in adults were mainly in the respiratory and cardiovascular systems, whereas the injuries in children were in the respiratory and nervous systems. The pathogenic characteristics in children are particularly similar to mouse model research (17). Although adult patients with SARS-CoV-2 infections presents a wide variety of neurological manifestations that range from mild to severe symptoms, such as anosmia, dysgeusia, myalgia, headache, hallucinations, psychomotor agitation, encephalopathy, vertigo, brain haemorrhage, brain ischaemia and encephalitis (27–29), the most common causes of death are ARDS, severe viral pneumonia, and multiple organ failure (30, 31). However, differences in pathogenicity between adults and children remain unknown. The mortality rates of hospitalised patients (in our study 9/659: 1.37%) in children are much lower than in adults (4.3%) (8), and in general, the younger the age, the milder the disease presentation.

The blood cell analysis had no typical features except the percentage of monocytes was slightly elevated. Among 659 inpatients patients, ALT was elevated in 17.91% of patients; AST was elevated in 49.01% of patients, and CK-MB were elevated in 46.28% of patients. This indicates that SARS-CoV-2 infection can cause multiple organ dysfunction (25, 26). PCT levels were elevated in 25.34% patients; however, this phenomenon is rare in viral infections, and the mechanism is not known. The limitations of this study are the short period of cases and the single-center study. The long-term 2019-ncov infection effects of children are still unclear, and further in-depth studies are urgently needed.

The Spearman's correlation analyses result show that children with SARS-CoV-2 Omicron Variant infection had higher levels of AST, ALT, CK-MB and PCT correlated with more risk of ANE and death. ALT, AST and CK-MB are important clinical indicators of disease severity. Regulated by endotoxins and cytokines, PCT may also be triggered by widespread tissue damage and endothelial dysfunction (32). The logistic analyses result showed that fever time upon admission (hour), peak fever, WBC, neutrophils percentage, CRP and convulsion frequency were independent risk factors for hospitalization of children with Omicron Variant infection. There is a relative paucity of pediatric data in the management of COVID-19 (33). Our research data will be useful for future research.

Summary, in the study, our findings is meaningful for further research of COVID-19 pathogenesis in children. The complex comorbidities, particularly nervous system damage are the clinical characteristic of pediatric patients with SARS-CoV-2 Omicron Variant infection. It is benefit to clinical management the severe coronavirus disease 2019 in in pediatric populations. Compare to previous pediatric COVID-19 literature, the same finding was neurologic diseases were significantly higher, with the high occurrence of seizures (34). But we analyzed the characteristics of the fatalities and showed imaging features of acute necrotizing encephalopathy. This study has several limitations. First, since this study was conducted in a limited number of big Children's hospital, there was potential selection bias. Second, due to the retrospective review of medical records, the judgment of the severity of illness was potentially misclassified and miss useful information. Finally, our data do not represent the national situation. Might prospective multicenter studies can analysis risk factors of the acute necrotizing encephalopathy, which lead main causes of death in pediatric patients with SARS-CoV-2 Omicron Variant infection. It is aim to provide a basis for early identification and treatment.

## 5 Conclusions

COVID-19 initially presents with “flu”-like symptoms. The most common symptom in children is a significant increase in the number of convulsions. If there is a high or ultra-high fever and progressive disturbance of consciousness, it is necessary to be alert to the occurrence of encephalitis, which can progress to life-threatening systemic inflammation and multi-organ dysfunction. Acute necrotising encephalopathy and pneumonia with comorbidities are the main causes of death in children with SARS-CoV-2 infection. High incidence of febrile seizures is a typical clinical characteristic. This indicates that nervous system damage in children with SARS-CoV-2 infection is more significant. It is significantly different from that in adults who die of respiratory distress syndrome (white lungs).

## Data Availability

The original contributions presented in the study are included in the article/Supplementary Material, further inquiries can be directed to the corresponding author.
